# Effects of temporal floral resource availability and non-crop habitats on broad bean pollination

**DOI:** 10.1007/s10980-022-01448-2

**Published:** 2022-04-30

**Authors:** Philipp W. Eckerter, Matthias Albrecht, Colette Bertrand, Erika Gobet, Felix Herzog, Sonja C. Pfister, Willy Tinner, Martin H. Entling

**Affiliations:** 1 iES Landau, Institute for Environmental Sciences, University of Koblenz-Landau, Landau, Germany; 2 Agricultural Landscapes and Biodiversity, Agroscope, Zurich, Switzerland; 3 Université Paris-Saclay, INRAE, UMR ECOSYS, AgroParisTech, Versailles, France; 4 Institute of Plant Sciences and Oeschger Centre for Climate Change Research, University of Bern, Bern, Switzerland; 5 Institute for Agroecology and Biodiversity (IFAB), Mannheim, Germany

**Keywords:** *Bombus*, Ecosystem services, Landscape composition, Crop pollination, *Vicia faba*, Wild bees

## Abstract

**Context:**

Flowering plants can enhance wild insect populations and their pollination services to crops in agricultural landscapes, especially when they flower before the focal crop. However, characterizing the temporal availability of specific floral resources is a challenge.

**Objectives:**

Developing an index for the availability of floral resources at the landscape scale according to the specific use by a pollinator. Investigating whether detailed and temporally-resolved floral resource maps predict pollination success of broad bean better than land cover maps.

**Methods:**

We mapped plant species used as pollen source by bumblebees in 24 agricultural landscapes and developed an index of floral resource availability for different times of the flowering season. To measure pollination success, patches of broad bean (*Vicia faba*), a plant typically pollinated by bumblebees, were exposed in the center of selected landscapes.

**Results:**

Higher floral resource availability before bean flowering led to enhanced seed set. Floral resource availability synchronous to broad bean flowering had no effect. Seed set was somewhat better explained by land cover maps than by floral resource availability, increasing with urban area and declining with the cover of arable land.

**Conclusions:**

The timing of alternative floral resource availability is important for crop pollination. The higher explanation of pollination success by land cover maps than by floral resource availability indicates that additional factors such as habitat disturbance and nesting sites play a role in pollination. Enhancing non-crop woody plants in agricultural landscapes as pollen sources may ensure higher levels of crop pollination by wild pollinators such as bumblebees.

**Supplementary information:**

The online version contains supplementary material available at 10.1007/s10980-022-01448-2.

## Introduction

Pollination by insects is crucial to reproduction for many plants. Animal pollination benefits 88% of wild flowering plants (Ollerton et al. 2011) and 35% of global crop production (Klein et al. 2007). The worldwide economic value of crop pollination in 2015 was estimated as 153 billion € (Gallai et al. 2009). During the last few decades, the dependency of global agriculture on pollinators has increased (Aizen et al. 2019). Wild insects greatly contribute to pollination in addition to managed bees (Garibaldi et al. 2013; Mallinger and Gratton 2015). Apart from wild bees, also flies, wasps, beetles and butterflies are frequently involved in crop pollination (Rader et al. 2016). Globally, roughly half of the economic value of crop pollination has been attributed to wild pollinators (Kleijn et al. 2015).

Agricultural intensification has led to declines in pollinator populations (IPBES 2016). Aside from pesticides and diseases, the scarcity of floral and nesting resources in agricultural landscapes are major causes of the decline in bee populations (IPBES 2016). Thus, the addition of flowering crop or non-crop plants to agricultural landscapes can enhance wild pollinators and their pollination of agricultural crops (Blaauw and Isaacs 2014; Venturini et al. 2017; Sutter et al. 2017, 2018; Ganser et al. 2018; Nicholson et al. 2019). The abundance and diversity of floral resources may lead to better crop pollination by supporting a higher diversity of bee pollinators (Potts et al. 2003; Fründ et al. 2010; Garibaldi et al. 2013; Blaauw and Isaacs 2014). In addition, the timing of the flowering of these alternative floral resources relative to the flowering period of crops may also be an important factor in their effect on crop pollination (Grab et al. 2017; Kremen et al. 2019). Availability of early flowering plants is expected to enhance pollinator populations and thus to benefit pollination of later flowering crops. For example, mass flowering oilseed rape (*Brassica napus*) facilitated later colony development of *Bombus terrestris* (Westphal et al. 2003, 2009) as well as later abundance of *Osmia bicornis* (Holzschuh et al. 2013). Mass flowering oilseed rape also enhanced bumblebee densities in later flowering sunflower crops (Riedinger et al. 2014) and pollination of wild shrubs in adjacent hedgerows (Kovács-Hostyánszki et al. 2013). Furthermore, mass-flowering apple trees successively led to higher pollination and yield of strawberries, most likely due to the increased abundance and diversity of bees present in the landscapes (Grab et al. 2017). Similarly, mass-flowering oilseed rape also led to higher yield in the later-flowering strawberry crop, given low proportions of semi-natural grassland in the surrounding landscape (Herbertsson et al. 2017). In contrast, plant species that produce high amounts of pollen and/or nectar synchronously with the focal crop may reduce crop pollination by competition (Lander et al. 2011; Bartomeus and Winfree 2011). For example, synchronous mass flowering oilseed rape reduced pollination of *Primula veris* in nearby calcareous grasslands due to shared bumblebee pollinators (Holzschuh et al. 2011) as well as reducing pollination of nearby wild shrubs (Kovács-Hostyánszki et al. 2013). Likewise, synchronous mass-flowering apple trees reduced pollinator activity and yield in strawberries (Grab et al. 2017). Visitation rate of pollinators for wild flowers and oilseed rape decreased when flower strips flowered synchronously in late June-late August (Häussler et al. 2017).

To account for the effects of alternative floral resources, it is necessary to characterize the availability of resources at different times of the season. However, this requires the mapping of the available floral resources across land cover types at the landscape level, which is a major challenge, and in the context of crop pollination such data has rarely been recorded. So far, studies of pollinators in agricultural landscapes distinguish only a small number of land cover types with different suitability for pollinators (Forman 1995; Fahrig 2013). Other studies included foraging distances to explain relative abundance of pollinators in nesting habitats (Lonsdorf et al. 2009) or combined foraging distances and resource quality to explain distribution of foraging bees (Olsson et al. 2015). Further, land cover classes and floral resources were used to explain colony growth and queen production of *Bombus vosnesenskii* (Crone and Williams 2016). Only recently, the temporal dynamics of focal and alternative floral resources were taken into account to explain crop visitation rates of wild pollinators (Häussler et al. 2017) or to explain the effects of a preceding versus a synchronous single mass flowering resource on crop pollination success (Grab et al. 2017).

In the present study, we combine a new method to quantify floral resource availability with a crop pollination experiment using broad bean (*Vicia faba* L.). The work was conducted in 24 landscapes selected along a gradient in the availability of preceding and synchronous alternative floral resources. We quantified floral resource availability at the species level across all major land cover types in the landscapes in combination with specific floral resource use information of crop pollinators. The broad bean is an insect-pollinated crop mostly pollinated by bumblebees (Kendall and Smith 1975; Stoddard and Bond 1987; Garratt et al. 2014). We inferred detailed information on floral resources from pollen types used by *Bombus terrestris*, one of the dominant bumblebee species, in different periods of the year and used an index to describe the availability of preceding and synchronous floral resources (Eckerter et al. 2020). In addition, we explored whether such temporally-resolved floral resource maps predict pollination better than land cover maps built on landscape characteristics such as the proportion of crops, forest, other semi-natural habitats or urban area. These floral resource maps are based on detailed, pollinator-specific and spatio-temporal information on floral resources that directly and evidently regulate pollinator populations (e.g. Roulston and Goodell 2011). We hypothesize that such floral resource maps predict the crop pollination performance of those pollinators better than land cover maps, which can only indirectly account for such information (Forman 1995; Roulston and Goodell 2011; Fahrig 2013; Eckerter et al. 2020; Ammann et al. 2022).

We tested the following hypotheses:

I. High availability of floral resources preceding crop flowering enhances pollination success.
II. High availability of alternative floral resources synchronous to crop flowering reduces pollination success.
III. Detailed floral resource maps predict crop pollination better than land cover maps.

## Methods

### Study design experimental set up

The study was conducted around the city of Landau in the Upper Rhine Valley, Rhineland-Palatinate, Germany. Broad bean (*Vicia faba* L. Var. Sutton Dwarf; Kings Seeds, Essex, UK) phytometers were exposed in the center of 24 study landscapes of 500 m radius (Online Resource, Fig. A.1). Landscapes were selected along gradients of dominant preceding (i.e. *Prunus* type, *Acer*, *Aesculus*, *Fragaria* and Brassicaceae) and synchronous (i.e. *Tilia*, *Rubus* and *Asparagus*) pollen resources used by bumblebees during the foraging season in the same region (Bertrand et al. 2019). While the broad bean is self-fertile, pollination from bees improves seed set (Aouar-sadli et al. 2008; Bartomeus et al. 2014; Nayak et al. 2015; Marzinzig et al. 2018), and bumblebees are among the main and most effective pollinators of this plant (Garratt et al. 2014; Bartomeus et al. 2014; Marzinzig et al. 2018).

For floral resource maps, we distinguished plant species flowering (1) preceding and (2) synchronous to broad beans. We considered 32 key pollen types that included all woody plants found to be used by *Bombus terrestris*, plus herbaceous plants representing more than 5% of pollen grains collected by the bumblebee at any point in time (i.e. either preceding or synchronous to broad bean flowering; data from Bertrand et al. 2019; Online Resource, Table A.1). We mapped the cover (m^2^) of all 69 plant species offering these 32 key pollen types in our study region between late May and November 2017 (Online Resource, A.1). Annuals (*Papaver rhoeas*, *Phacelia tanacetifolia* and *Trifolium* spec.) were mapped during their flowering period (late-May until mid-July) in all landscapes. The pollen collected from all of the mapped plant species accounted for 84% of the pollen diet of *Bombus terrestris* across the season (Bertrand et al. 2019). The unmapped plant species, which made up the 39 remaining pollen types identified as part of bumblebees’ diets in Bertrand et al. (2019) but were not included in this study, were mostly herbaceous plants with relatively low floral abundances (Online Resource, Table A.2). For land cover maps, all arable land, permanent crops, forest, other woody semi-natural habitat, herbaceous semi-natural habitat and urban areas were mapped in all landscapes according to field inspection and aerial photographs. Landscapes consisted mainly of crops (average: 70%, range: 29–97%) and herbaceous semi-natural habitat (average: 11%, range: 1–51%). Main crops were cereals, maize and sugar beet.

Bumblebees forage mostly within a radius of 500 m around their colony, although longer foraging flights are possible (Osborne et al. 1999; Kreyer et al. 2004; Wolf and Moritz 2008). In order to keep landscape gradients as independent as possible from each other, landscape centers were separated from each other by at least 800 m (average: 10,391 m, standard error: 252 m). All landscape centers were located in grassy field margins. Twenty pots with one plant of broad bean *Vicia faba* L. var. Sutton Dwarf each were exposed in each landscape center (Online Resource, Fig. A.2). The plants were grown in greenhouses and net cages with no pollinator access before or after field exposure. When sowing the beans, we applied 1000 g of NPK 6-17-27 fertilizer per m^3^ of soil. The pots with full flowering plants (BBCH65; Lancashire et al. 1991) were watered regularly and placed in two rows with a distance of 0.3 m between pots and 0.5 m between rows. The segments of plants that only flowered during field exposure were marked with cable ties and later evaluation of pollination success was restricted to flowers of these segments. Two independent sets of plants were exposed in the field, one from 25th May to 9th June and the other from 13th to 28th June 2017. Both exposure periods occurred after the flowering of the major early pollen sources in the study region such as *Acer*, *Aesculus*, *Brassicaceae* (mainly oilseed rape), *Crataegus*, *Fragaria*, *Prunus* and *Salix* but simultaneous to major late-flowering pollen resources such as *Papaver*, *Phacelia*, *Rubus* and *Tilia* in order to reflect the typical flowering time of *Vicia faba* in the study region. To verify the general role of insect pollination on seed set of the used variety, we placed one additional plant per landscape center into a gauze cage (“Aerarium Size L”, Aerarium Nets GmbH, Switzerland, 155 meshes per cm^2^) next to the other sentinels. To obtain an overview of flower visitors, we employed camcorders (Sony HDR-CX115E) once during the morning (in between 8.45 and 11.30 am) and once during the afternoon (in between 3.30 and 5.30 pm), for 1:50 h each and a total of 3:40 h of video observation in each landscape. After returning all plants to the greenhouse, they were watered every two days until early August, when pods were fully ripe (BBCH89; Lancashire et al. 1991). Two weeks later, the dried pods were harvested. Pods and seeds were counted in the lab.

### Data analysis

To test our hypotheses, we used the number of seeds per pod as an indicator of pollination success as open pollination led to higher seed set in other studies (Free 1966; Ishag 1973; Suso et al. 1996; Aouar-sadli et al. 2008; Nayak et al. 2015).

The cover of different land use types and the distribution of plant species providing pollen resources were digitized as vector layers and analyzed with the geographic information system QGIS V. 3.6 (QGIS Development Team 2018; Online Resource, Table A.2).

The availability of different floral resources in each landscape during a time period was combined into a floral resource availability index (*fai)* that weighed the relative cover of each flowering plant species in a landscape (*cr*_*p,l*_) by its quantitative utilization by workers of *B. terrestris* in our study region (*vr*_*p,t*_; Eq. 1; see below for details; Bertrand et al. 2019). These indices were calculated for each landscape *l* for three time periods *t*: (1) preceding broad bean exposure (i.e. start of flowering season in mid-March until late May), (2) synchronous to broad bean exposure (i.e. late May to late June) and (3) pooled across the whole study period. To account for the range in preference of different pollen sources to *B. terrestris*, we used pollen volume collected by multiple colonies across multiple landscapes in our study region (Bertrand et al. 2019) as a proxy of preference. The total cover of plants providing each pollen type across all landscapes was, thus, weighted proportionally to the total pollen volume of each time period. This ensured that the contribution of each plant type to the pollen availability index was proportional to the preference of this plant type for bumblebees (e.g. a plant type accounting for 20% of pollen use by *Bombus* counts ten times more than a plant type accounting for 2% of pollen use).

1 $$fa{i_{l,t}} = n \cdot \sum\limits_{p = 1}^P {c{r_{p,l}} \cdot v{r_{p,t}}}$$

In this equation, *n* represents the number of landscapes, *P* is the number of key pollen types flowering in the respective time period, *cr*_*p,l*_ is the cover of plants providing pollen type *p* in the respective landscape *l* divided by their total cover across all landscapes and *vr*_*p,t*_ is the volume of pollen type *p* in the diet of *Bombus terrestris* divided by the volume of all pollen recorded in their diet during the respective time period *t*. This index returns a positive decimal value, whereby a value of 1 corresponds to the average pollen availability across all landscapes at the respective time. Values below 1 indicate below-average pollen availability, whereas values higher than 1 reveal an above-average pollen availability. For more details on the index see Online Resource, A.2. Whenever we use the term “floral resources” in the remainder of this paper, we are referring to the resource availability index *fai*.

For the land cover maps approach, landscape context was expressed as the proportion of arable land, permanent crops, forest, other woody semi-natural habitat, herbaceous semi-natural habitat and urban areas in the landscape. Euclidean distances from the broad bean sentinels in the landscape centers to the nearest forest or urban land use were also calculated.

To test and compare predictability of seed set by the two mapping approaches, a model containing all explanatory variables was set up for each approach. To facilitate interpretation of parameter estimates, input variables were standardized by dividing by two standard deviations using the *standardize* function from the *arm* package (Gelman 2008). Models of each mapping approach were compared based on Akaikes second-order information criterion for small sample sizes (AICc; Akaike 1987; Burnham et al. 2011; Hurvich and Tsai 1989; Symonds and Moussalli 2011) using the *dredge* function from the *MuMin* package (Barton 2020) and a cutoff rule (Δ_*i*_ < 2) (Burnham and Anderson 2002; Symonds and Moussalli 2011). For comparison of seed set predictability of both mapping approaches, *R*^*2*^_*mult*_ and *R*^*2*^_*adj*_ values for the most parsimonious models were compared. Effects of landscape context on seed set were assessed using models from the subset of models best explaining seed set (i.e. all models with (Δ_*i*_ < 2). Contributions of landscape context to floral resource availability were assessed with linear regression models. Linear models were plotted using the package *ggplot2* (Wickham 2016). In order to determine whether land cover maps would be more effective when using finer categories (i.e. division of crops into the classes of arable land and permanent crops as well as semi-natural habitat into the classes forest, other woody and herbaceous semi-natural habitats), their performance in predicting seed set and contributions of landscape context to floral resource availability were compared using linear regression. All statistical analyses were conducted using *R* 4.0 (R Core Team 2020). Diagnostic plots (residuals vs. fitted values and normal Q–Q plots) were visually checked. We further assessed correlations among explanatory variables and created a correlation plot using the *corrplot* package in *R* (Wei and Simko 2017).

## Results

The pollen use by *Bombus terrestris* during the various time periods is shown in Online Resource, Table A.1. From 55,099 broad bean flowers, we harvested 1,328 pods (mean per landscape = 55.3 ± 14.3) with at least one developed seed and 3,269 (mean = 136.2 ± 37.2) developed seeds in total. The mean number of developed seeds per pod per landscape ranged from 2 to 2.8 (mean = 2.5 ± 0.2). The caged plants developed no seeds. The video observations recorded the bumblebee species *B. terrestris* agg. *(B. terrestris*, *B. lucorum*, *B. cryptarum* and *B. magnus*, n = 25), *B. hortorum* (n = 11) and *B. lapidarius* (n = 1) as well as the honeybee *Apis mellifera* (n = 34) as pollinators of the sentinel plants.

### Floral resource maps

**Fig. 1:**
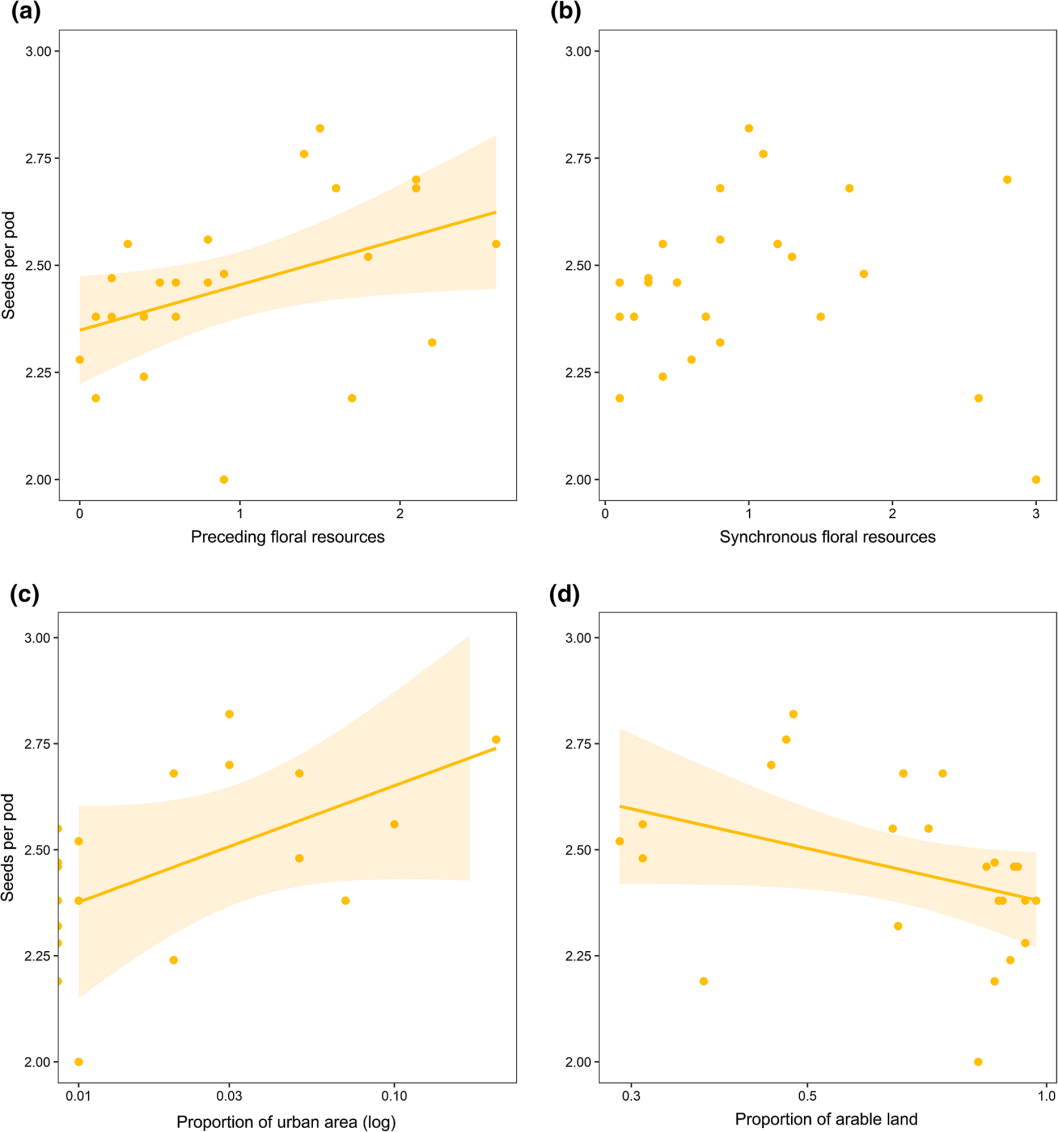
Relationships between seed set and floral resource availability **a** preceding broad bean (*Vicia faba* L.) flowering, **b** synchronous to broad bean flowering as well as relationships with proportions of **c** urban area and **d** arable land. Predicted linear relationships and 95% confidence intervals are shown for statistically significant results (**a**, **c** and **d**)

As expected, broad bean seed set increased in landscapes with high preceding floral resource availability (t_1,22_ = 2.19, *R*^*2*^_*mult*_ = 0.18, p = 0.039, Fig. 1a). In contrast, synchronous floral resources had no significant influence on seed set (t_1,22_ = − 0.26, *R*^*2*^_*mult*_ < 0.01, p = 0.797, Fig. 1b). Floral resources pooled across the whole season had no significant influence on seed set of broad beans (t_1,22_ = 0.74, *R*^*2*^_*mult*_ = 0.02, p = 0.466).

### Land cover maps

In the best models based on land cover, seed set increased with urban area in the landscape (t_1,21_ = 2.89, *R*^*2*^_*adj*_ = 0.26, p < 0.01; Fig. 1c). In alternative models containing the proportion of arable land (Δ_*i*_ **≥** 1.7), seed set decreased with increasing proportion of arable land (t_1,20_ = 2.25, *R*^*2*^_*adj*_ = 0.27, p = 0.036, Fig. 1d). Correlations of seed set and other landscape variables contained in these models were non-significant (p > 0.05; Table 1).

**Table 1:** Comparison of selected models best explaining seed set of broad beans with linear regression using the mapping approaches of floral resource maps and land cover maps. Model selection is based on Akaikes second-order Information Criterion (AICc). Only models considered to be as good as the most parsimonious model (i.e. Δ_i_ < 2) are shown. R^2^_mult_ and R^2^_adj_ are the proportions of variances explained by models using one or more variables, respectively. Delta weight Δ_i_ is the difference between the AICc for a model and the most parsimonious model, and Akaike weight w_i_ is the probability that the model i is the most parsimonious model of models given. To make estimates and standard errors comparable between the models, variables were standardized by dividing by two standard deviations. Significant relations (p < 0.05) are printed in bold

Method	Model description	df	*R*^*2*^_*mult*_	*R*^*2*^_*adj*_	AICc	Δ_*i*_	w_*i*_	Predictor	Estimate	SE	t value	p value
Floral resources	Preceding	22	0.179	0.142	− 8.6	0.00	0.62	**Preceding**	**0.1661**	**0.0759**	**2.19**	**0.039**
	Preceding + Synchronous	21	0.241	0.169	− 7.6	1.01	0.38	**Preceding**	**0.2099**	**0.0818**	**2.57**	**0.018**
								Synchronous	− 0.1074	0.0818	− 1.31	0.203
Land cover maps	Crop permanent + Urban	21	0.324	0.259	− 10.3	0.00	0.25	Crop permanent	0.1394	0.0723	1.93	0.068
								**Urban**	**0.2088**	**0.0723**	**2.89**	**0.009**
	Crop permanent + Forest + Urban	20	0.390	0.299	− 9.6	0.73	0.18	Crop permanent	0.1415	0.0704	2.01	0.058
								Forest	0.1015	0.0686	1.48	0.154
								**Urban**	**0.2080**	**0.0704**	**2.96**	**0.008**
	Urban	22	0.204	0.168	− 9.3	1.00	0.15	**Urban**	**0.1774**	**0.0747**	**2.37**	**0.027**
	Arable + Herbaceous SNH + Urban	20	0.366	0.271	− 8.6	1.69	0.11	**Arable**	**− 0.2382**	**0.1061**	**− 2.25**	**0.036**
								Herbaceous SNH	− 0.1812	0.1054	− 1.72	0.101
								Urban	0.1513	0.0777	1.95	0.066
	Arable	22	0.179	0.142	− 8.6	1.73	0.11	**Arable**	**− 0.1664**	**0.0758**	**− 2.19**	**0.039**
	Arable + Urban	21	0.272	0.203	− 8.6	1.77	0.11	Arable	− 0.1123	0.0803	− 1.40	0.176
								Urban	0.1310	0.0803	1.63	0.118
	Forest + Urban	21	0.267	0.198	− 8.4	1.92	0.10	Forest	0.0988	0.0734	1.35	0.192
								**Urban**	**0.1761**	**0.0734**	**2.40**	**0.026**

Seed set was somewhat better predicted using land cover maps (*R*^*2*^_*adj*_ = 0.26) compared to floral resource maps (*R*^*2*^_*mult*_ = 0.18) according to the respective most parsimonious model of each mapping approach (Δ_*i*_ **=** 1.7; Table 1).

### Contribution of land cover to floral resource availability

Floral resources preceding crop flowering were negatively correlated with the proportion of arable land in the landscape (t_1,22_ = − 3.55, *R*^*2*^_*mult*_ = 0.364, p < 0.01). They increased with the proportion of permanent crops (t_1,22_ = 2.20, R^2^ = 0.181, p = 0.038) and woody semi-natural habitat other than forest (t_1,22_ = 2.11, *R*^*2*^_*mult*_ = 0.168, p = 0.047).

Synchronous floral resources were negatively correlated with the proportion of arable land (t_1,22_ = − 2.88, *R*^*2*^_*mult*_ = 0.274, p < 0.01) and distance to forest (t_1,22_ = − 3.08, *R*^*2*^_*mult*_ = 0.301, p = 0.006). They increased with the proportion of forest (t_1,22_ = 3.12, *R*^*2*^_*mult*_ = 0.307, p = 0.005). For complete correlations among variables see Online Resource Table A.3 and Fig. A.3. For a complete list of regression models between seed set and landscape context see Online Resource Table A.4. When using land cover maps, the division of broad habitat categories (crop and semi-natural habitat) into the finer categories of arable land and permanent crops as well as forest, other woody and herbaceous semi-natural habitat improved predictability of seed set (*R*^*2*^_*mult*_ = 0.140 compared to *R*^*2*^_*mult*_ = 0.179 for broad and fine resolution, respectively; Online Resource Table A.5).

Wild plants contributed more to floral resource availability preceding and synchronous to broad bean flowering (72% and 95%, respectively) than cultivated plants. Regarding their vegetation type (i.e. either herbaceous or woody), woody plant types contributed more to floral resource availability (preceding: 94%, synchronous: 76%) than herbaceous plants.

## Discussion

### Temporal floral resource maps

As predicted, pollination success of broad bean increased with the availability of preceding floral resources in the landscapes. This confirms our first hypothesis that increasing pollinator populations early in the year lead to higher pollinator visitation of subsequently flowering crops. These findings are similar to Grab et al. (2017), who observed that preceding mass-flowering apple enhanced successive strawberry pollination. Our results show that the timing of alternative floral resources is also crucial in more diverse landscapes, where a high number of plant species provide alternative resources to key pollinators. By accounting for the varied pollen usage of *Bombus terrestris* in our study region (Bertrand et al. 2019), we could show that the presence of fruit trees (*Prunus* spec.), maple (*Acer* spec.) and willow (*Salix* spec.) contribute to this higher flower availability for *B. terrestris* in the early season. The combined contribution of these three groups of trees to floral resource availability for *Bombus terrestris* preceding broad bean flowering was 75%.

In contrast, the availability of synchronous alternative resources during broad bean flowering had neither a positive nor a negative effect on pollination success. This contrasts with the decline of early-flowering strawberry pollination with increasing synchronous mass-flowering apple observed by Grab et al. (2017). This difference could be explained by contrasting attractiveness of the focal crop versus the alternative resources to pollinators between the two studies. According to Abrol (1990, 1992), strawberry plants have a comparatively low attractivity for pollinators in contrast to mass-flowering apple based on higher total daily energy reward per apple flower compared to that of strawberry. Additionally, flower density is higher in apple orchards than in strawberry fields. Therefore, it is not surprising that apple attracts pollinators away from strawberry crops during simultaneous flowering. Conversely, broad bean is highly attractive, especially in terms of nectar, which could explain why its visitation did not significantly decline with increasing availability of synchronous alternative flowers such as *Papaver*, *Phacelia* and *Rubus*. Negative effects of synchronous flowering resources on yield due to competition in the late season might also be mitigated by positive carry-over effects of flowering resources from the preceding year. For example, late flowering resources in the landscapes not only led to an increased production of queens and males of bumblebees during the same year, but also to a higher density and species richness of bumblebee foragers in the following years (Rundlöf et al. 2014; Kallioniemi et al. 2017; Häussler et al. 2017). Thus, even if late alternative resources attract pollinators away from the focal crop, they may facilitate higher overall pollinator populations over time, which could lead to a net neutral effect on crop pollination. In addition, attractive synchronous flowering plants may have led to pollinator attraction from the wider landscape into the area where the broad beans were placed and, therefore, facilitated pollination (Morandin and Kremen 2013). This effect might have been higher for the smaller number of floral resources provided by phytometer plants compared to that provided by mass-flowering cultures. Hence, positive and negative effects of late floral resources may level each other out, which could explain why neither a positive nor a negative effect of synchronous floral resources on pollination was observed in our study. Of course, the dominance of either the negative effect via competition for pollinators or the positive effect of pollinator enhancement may also depend on the design and location of the study. We hypothesize that positive carry-over effects are more likely in landscapes in which other resources for pollinators such as nesting sites are not limited. In contrast, competition for pollinators is more likely in highly simplified landscapes, in which there are generally fewer pollinators and the alternative floral resources are highly attractive relative to the crop.

The video observations showed more individuals of *A. mellifera* collecting pollen on *Vicia faba* than of *B. terrestris*. Yet, in contrast to the bumblebees, honeybees showed a much calmer foraging behavior. *Bombus terrestris* has the ability to buzz pollinate, which enhances self-pollination by releasing pollen from the stigmas during the pollination process (“Tripping”; e.g. Pazy 1984). *Bombus terrestris* therefore contributes more to insect induced self-pollination than *A. mellifera* (De Luca & Vallejo-Marin 2013), even when flower visits are somewhat less frequent. We thus assume that *B. terrestris* is the main responsible for the seed set during our study.

### Land cover maps

Seed set increased with the proportion of urban area and decreased with arable land area recorded land cover maps. Indeed, pollinators can benefit from urban sprawl in city margins due to higher amounts of floral or nesting resources or benefits by intermediate levels of disturbance (Wenzel et al. 2020). Furthermore, urbanization leads to an increase of pollinator diversity compared to intensified agricultural areas, although diversity decreases in urban areas compared to natural or semi-natural areas (Wenzel et al. 2020). Colonies of *Bombus terrestris* were found to have developed more in suburban than agricultural areas due to a higher diversity and density of floral resources provided by gardens compared to farmland (Goulson et al. 2002). However, in our study, urban area was not significantly associated with flower availability (preceding, synchronous and total: r = 0.13, 0.08, 0.11, respectively). Nevertheless, pollinators may have benefited from other factors provided by urban areas such as nesting sites or reduced disturbance (i.e. offering shelter during adverse weather conditions or reduced application of agrochemicals), which were not documented in our study. Moreover, urban areas could have had higher densities of domestic honey bees, which might have enhanced field bean seed set. The observed negative effects of intensive agriculture on pollinators are consistent with literature (Goulson et al. 2015; Kovács-Hostyánszki et al. 2017; Pfister et al. 2018). The lack of a better predictive power of our maps based on the detailed floral resource availability suggests that floral resources alone are not the dominant factor limiting broad bean pollination in our study region. Other factors such as the availability of nesting habitat, disturbance of agricultural soils or pesticide applications may have been relevant. The effect of floral resource availability cannot clearly be distinguished from the effects of landscape composition because the statistical strength of the predictors was similar and the availability of preceding floral resources declined with the proportion of arable land (r = − 0.60) but not with the proportion of urban areas (r = 0.13).

Semi-natural habitat can positively affect wild bees and their performance in agricultural landscapes (Rollin et al. 2013, 2015; Crone and Williams 2016). Loss of semi-natural habitats or increasing distance between these habitats can have direct negative consequences on pollinators (Ricketts et al. 2008; Winfree et al. 2011) and, thus, on crop pollination as well (Kremen et al. 2004; Greenleaf and Kremen 2006; Klein et al. 2012). In our study, although pooled floral resources increased with proportion of herbaceous (r = 0.45) and woody semi-natural habitat other than forest (r = 0.42) in the landscapes, semi-natural habitats did not explain seed set. Additionally, Westphal et al. (2003) found densities of *Bombus terrestris* to be explained by the amount of mass-flowering oilseed rape rather than by semi-natural habitat. However, this crop is comparatively rare in the region where our study was conducted. There, woody semi-natural habitat included patches offering high amounts of floral resources, especially hedgerows and semi-natural orchards. Herbaceous semi-natural habitat, in contrast, contained wide areas of grassland under various management schemes with a rather low flower availability to bumblebees. Nevertheless, herbaceous semi-natural habitats could have provided nesting sites to bumblebees in the form of vole burrows, which were recently suggested to increase bee populations and crop visitation (Nicholson et al. 2019). In order to be beneficial for pollinators, semi-natural habitat may need to offer a minimum number of floral resources (Rollin et al. 2013, 2019). The dominance of pollen collected from woody plants in the diet of *Bombus terrestris* seen in our study aligns with earlier studies highlighting the importance of early-flowering trees and shrubs for bumblebees (e.g. Kämper et al. 2016). We observed higher floral availability synchronous to broad bean flowering and pooled across the whole season with increased amount and proximity to forest. This is partially explained by availability of wild-growing *Rubus*, contributing 31.7% to synchronous and 19% to pooled pollen use (Table A.1), which increased with forest (r = 0.65) and its proximity (r = 0.51) to the landscape centers. Pollination and bee abundance are shown to benefit from proximity to forest patches that also offer mating and nesting sites as well as nesting material (Bailey et al. 2014, and references therein). Proximity to other fields of *Vicia faba* in the surrounding landscape may increase cross-fertilization of plants thus leading to higher seed set. Considering the mean foraging range of *B. terrestris*, which mostly lies below 500 m (Osborne et al. 1999; Wolf and Moritz 2008), we inspected the 1,000 m radius around our landscape centers for close-by fields of *V. faba*. We recorded one single field of *V. faba* 880 m from one of our study sites. Seed set in that site was below average across all landscapes, with plants in 19 out of the 24 landscapes developing a higher seed set. We, therefore, assume that surrounding fields of *V. faba* did not critically lead to a higher cross-fertilization in our sentinels.

### Comparison of mapping approaches

In our study, seed set was somewhat better predicted by land cover maps than by floral resource maps, although the latter are based on detailed information that more directly and evidently relates to pollinator populations (Roulston and Goodell 2011). Similarly, land cover maps explained abundance of aphid predators and related aphid pest control on broad bean better than temporal floral resource maps in Switzerland (Ammann et al. 2022). This may be due to other above-mentioned parameters that were not assessed in this study (e.g. availability of nesting habitat) but which were likely important for pollinator activity. In addition, the floral resources were closely related to the land cover types used in this study. The predictive power of land cover maps improved when using finer habitat categories. Hence, connecting resources to finer habitat categories might help to further improve the prediction of pollinators with land cover maps and their use in conservation planning. Woody non-crop plants in the agricultural landscape such as hedgerows, woodlots and tree rows have been found to provide the highest densities of floral resources to bumblebees (Eckerter et al. 2020). In this study, exposed colonies of *B. terrestris* showed increased weight gain, queen production and survival with proximity to forest, although species-specific floral resource availability did not show significant effects on colonies. Beneficial effects of woody structures next to resource availability such as protection from adverse weather conditions or nectar provision may also play a role in directing pollinator activities in agricultural landscapes. As woody structures are also key for the conservation of farmland birds and predatory arthropods (e.g. Mestre et al. 2018), adding woody structures to agricultural landscapes would likely benefit overall biodiversity and other ecosystem services in addition to pollinators (e.g. Holland et al. 2017; Schirmel et al. 2017; Bartual et al. 2019). To transfer our findings to the crop level and relate them to food stability and production of agricultural systems further studies are needed. Such studies could address other responses of *Vicia faba* to pollinators (e.g. seed size) in relation with the spatio-temporal availability of floral resources in the surrounding landscape or consider crops other than *Vicia faba*.

### Conclusions and implications

Our study underlines the key role of early flowering resources for crop pollination in agricultural landscapes. However, the detailed examination of pollen types and their spatial and temporal availability in the landscapes did not allow for a clearer explanation of pollination success than simple landscape metrics such as the proportion of arable land. Further research may help to disentangle the effects that are combined in these simplified predictors. As most of the early-flowering resources were provided by wild trees and shrubs, flower-rich woody structures such as hedgerows and forest edges should be conserved in agricultural landscapes to ensure high levels of crop pollination by wild pollinators such as bumblebees.

## Supplementary Information

Below is the link to the electronic supplementary material.

Supplementary material 1 (DOCX 1583.7 kb)

## Data Availability

The data that support the findings of this study is available in figshare (10.6084/m9.figshare.12444707).

## References

[CR1] Abrol DP (1990). Energetics of nectar production in some apple cultivars as a predictor of floral choice by honeybees. Trop Ecol.

[CR2] Abrol DP (1992). Energetics of nectar production in some strawberry cultivars as a predictor of floral choice by honeybees. J Biosci.

[CR3] Aizen MA, Aguiar S, Biesmeijer JC (2019). Global agricultural productivity is threatened by increasing pollinator dependence without a parallel increase in crop diversification. Glob Change Biol.

[CR4] Akaike H (1987). Factor analysis and AIC. Psychometrika.

[CR5] Ammann L, Bosem-Baillod A, Eckerter PW (2022). Comparing floral resource maps and land cover maps to predict predators and aphid suppression on field bean. Landsc Ecol.

[CR6] Aouar-sadli M, Louadi K, Doumandji S-E (2008) Pollination of the broad bean (*Vicia faba* L. var. major) (Fabaceae) by wild bees and honey bees (Hymenoptera: Apoidea) and its impact on the seed production in the Tizi-Ouzou area (Algeria).Afr J Agric Res266–272

[CR7] Bailey S, Requier F, Nusillard B (2014). Distance from forest edge affects bee pollinators in oilseed rape fields. Ecol Evol.

[CR8] Bartomeus I, Winfree R (2011). The Circe Principle: Are pollinators waylaid by attractive habitats?. Curr Biol.

[CR9] Bartomeus I, Potts SG, Steffan-Dewenter I (2014). Contribution of insect pollinators to crop yield and quality varies with agricultural intensification. PeerJ.

[CR10] Barton K (2020) MuMIn: Multi-Model Inference. Version R package version 1.43.17 https://CRAN.R-project.org/package=MuMIn

[CR89] Bartual Agustín M., Sutter Louis, Bocci Gionata, Moonen Anna-Camilla, Cresswell James, Entling Martin, Giffard Brice, Jacot Katja, Jeanneret Philippe, Holland John, Pfister Sonja, Pintér Orsolya, Veromann Eve, Winkler Karin, Albrecht Matthias (2019). The potential of different semi-natural habitats to sustain pollinators and natural enemies in European agricultural landscapes. Agriculture, Ecosystems & Environment.

[CR11] Bertrand C, Eckerter PW, Ammann L (2019). Seasonal shifts and complementary use of pollen sources by two bees, a lacewing and a ladybeetle species in European agricultural landscapes. J Appl Ecol.

[CR12] Blaauw BR, Isaacs R (2014). Flower plantings increase wild bee abundance and the pollination services provided to a pollination-dependent crop. J Appl Ecol.

[CR13] Burnham KP, Anderson DR (2002). Model selection and multimodel inference: a practical information-theoretic approach.

[CR14] Burnham KP, Anderson DR, Huyvaert KP (2011). AIC model selection and multimodel inference in behavioral ecology: some background, observations, and comparisons. Behav Ecol Sociobiol.

[CR15] Crone EE, Williams NM (2016). Bumble bee colony dynamics: quantifying the importance of land use and floral resources for colony growth and queen production. Ecol Lett.

[CR16] De Luca PA, Vallejo-Marín M (2013). What’s the ‘buzz’ about? The ecology and evolutionary significance of buzz-pollination. Curr Opin Plant Biol.

[CR17] Eckerter PW, Albus L, Natarajan S, Albrecht M, Ammann L et al (2020) Using temporally resolved floral resource maps to explain bumblebee colony performance in agricultural landscapes. Agronomy 10:1993

[CR18] Fahrig L (2013). Rethinking patch size and isolation effects: the habitat amount hypothesis. J Biogeogr.

[CR19] Forman RTT (1995). Land Mosaics: The Ecology of Landscapes and Regions.

[CR20] Free JB (1966). The pollination requirements of broad beans and field beans (*Vicia faba*). J Agric Sci.

[CR21] Fründ J, Linsenmair KE, Blüthgen N (2010). Pollinator diversity and specialization in relation to flower diversity. Oikos.

[CR22] Gallai N, Salles J-M, Settele J, Vaissière BE (2009). Economic valuation of the vulnerability of world agriculture confronted with pollinator decline. Ecol Econ.

[CR23] Ganser D, Mayr B, Albrecht M, Knop E (2018). Wildflower strips enhance pollination in adjacent strawberry crops at the small scale. Ecol Evol.

[CR24] Garibaldi LA, Steffan-Dewenter I, Winfree R (2013). Wild pollinators enhance fruit set of crops regardless of honey bee abundance. Science.

[CR25] Garratt MPD, Coston DJ, Truslove CL (2014). The identity of crop pollinators helps target conservation for improved ecosystem services. Biol Conserv.

[CR26] Gelman A (2008). Scaling regression inputs by dividing by two standard deviations. Statist Med.

[CR27] Goulson D, Hughes W, Derwent L, Stout J (2002). Colony growth of the bumblebee, *Bombus terrestris*, in improved and conventional agricultural and suburban habitats. Oecologia.

[CR28] Goulson D, Nicholls E, Botias C, Rotheray EL (2015). Bee declines driven by combined stress from parasites, pesticides, and lack of flowers. Science.

[CR29] Grab H, Blitzer EJ, Danforth B (2017). Temporally dependent pollinator competition and facilitation with mass flowering crops affects yield in co-blooming crops. Sci Rep.

[CR30] Greenleaf SS, Kremen C (2006). Wild bees enhance honey bees’ pollination of hybrid sunflower. Proc Natl Acad Sci USA.

[CR31] Häussler J, Sahlin U, Baey C (2017). Pollinator population size and pollination ecosystem service responses to enhancing floral and nesting resources. Ecol Evol.

[CR32] Herbertsson L, Rundlöf M, Smith HG (2017). The relation between oilseed rape and pollination of later flowering plants varies across plant species and landscape contexts. Basic Appl Ecol.

[CR33] Holzschuh A, Dormann CF, Tscharntke T, Steffan-Dewenter I (2011). Expansion of mass-flowering crops leads to transient pollinator dilution and reduced wild plant pollination. Proc R Soc B.

[CR34] Holzschuh A, Dormann CF, Tscharntke T, Steffan-Dewenter I (2013). Mass-flowering crops enhance wild bee abundance. Oecologia.

[CR87] Holland JM, Douma JC, Crowley L, James L, Kor L et al (2017) Semi-natural habitats support biological control pollination and soil conservation in Europe. A review. Agron Sustain Dev 37(4):31

[CR35] Hurvich CM, Tsai C-L (1989) Regression and time series model selection in small samples.Biometrika 297–307

[CR36] IPBES (2016). The assessment report of the Intergovernmental Science-Policy Platform on Biodiversity and Ecosystem Services on pollinators, pollination and food production.

[CR37] Ishag HM (1973). Physiology of seed yield in field beans (*Vicia faba* L.): I. Yield and yield components. J Agric Sci.

[CR38] Kallioniemi E, Åström J, Rusch GM (2017). Local resources, linear elements and mass-flowering crops determine bumblebee occurrences in moderately intensified farmlands. Agr Ecosyst Environ.

[CR39] Kämper W, Werner PK, Hilpert A (2016). How landscape, pollen intake and pollen quality affect colony growth in *Bombus terrestris*. Landsc Ecol.

[CR40] Kendall DA, Smith BD (1975). The pollinating efficiency of honeybee and bumblebee visits to field bean flowers (*Vicia faba* L.). J Appl Ecol.

[CR41] Kleijn D, Winfree R, Bartomeus I (2015). Delivery of crop pollination services is an insufficient argument for wild pollinator conservation. Nat Commun.

[CR42] Klein A-M, Vaissière BE, Cane JH (2007). Importance of pollinators in changing landscapes for world crops. Proc R Soc B.

[CR43] Klein A-M, Brittain C, Hendrix SD (2012). Wild pollination services to California almond rely on semi-natural habitat: Wild pollination services to California almond. J Appl Ecol.

[CR44] Kovács-Hostyánszki A, Haenke S, Batáry P (2013). Contrasting effects of mass-flowering crops on bee pollination of hedge plants at different spatial and temporal scales. Ecol Appl.

[CR45] Kovács-Hostyánszki A, Espíndola A, Vanbergen AJ (2017). Ecological intensification to mitigate impacts of conventional intensive land use on pollinators and pollination. Ecol Lett.

[CR46] Kremen C, Williams NM, Bugg RL (2004). The area requirements of an ecosystem service: Crop pollination by native bee communities in California: Area requirements for pollination services to crops. Ecol Lett.

[CR47] Kremen C, Albrecht M, Ponisio LC, Dover JW (2019). Restoring pollinator communities and pollination services in hedgerows in intensively-managed agricultural landscapes. The Ecology of Hedgerows and Field Margins.

[CR48] Lancashire PD, Bleiholder H, Boom TVD (1991). A uniform decimal code for growth stages of crops and weeds. Ann Appl Biol.

[CR49] Lander TA, Bebber DP, Choy CTL (2011). The Circe Principle explains how resource-rich land can waylay pollinators in fragmented landscapes. Curr Biol.

[CR50] Lonsdorf E, Kremen C, Ricketts T (2009). Modelling pollination services across agricultural landscapes. Ann Bot-London.

[CR51] Mallinger RE, Gratton C (2015). Species richness of wild bees, but not the use of managed honeybees, increases fruit set of a pollinator-dependent crop. J Appl Ecol.

[CR52] Marzinzig B, Brünjes L, Biagioni S (2018). Bee pollinators of faba bean (*Vicia faba* L.) differ in their foraging behaviour and pollination efficiency. Agr Ecosyst Environ.

[CR53] Mestre L, Schirmel J, Hetz J (2018). Both woody and herbaceous semi-natural habitats are essential for spider overwintering in European farmland. Agr Ecosyst Environ.

[CR54] Morandin LA, Kremen C (2013). Hedgerow restoration promotes pollinator populations and exports native bees to adjacent fields. Ecol Appl.

[CR55] Nayak GK, Roberts SPM, Garratt M (2015). Interactive effect of floral abundance and semi-natural habitats on pollinators in field beans (*Vicia faba*). Agr Ecosyst Environ.

[CR56] Nicholson CC, Ricketts TH, Koh I (2019). Flowering resources distract pollinators from crops: Model predictions from landscape simulations. J Appl Ecol.

[CR57] Ollerton J, Winfree R, Tarrant S (2011). How many flowering plants are pollinated by animals?. Oikos.

[CR58] Olsson O, Bolin A, Smith HG, Lonsdorf EV (2015). Modeling pollinating bee visitation rates in heterogeneous landscapes from foraging theory. Ecol Model.

[CR85] Osborne J.L., Clark S.J., Morris R.J., Williams I.H., Riley J.R., Smith A.D., Reynolds D.R., Edwards A.S. (1999). A landscape-scale study of bumble bee foraging range and constancy, using harmonic radar. J Appl Ecol.

[CR59] Pazy B (1984). Insect Induced Self-Pollination. Pl Syst Evol.

[CR60] Pfister SC, Eckerter PW, Krebs J (2018). Dominance of cropland reduces the pollen deposition from bumble bees. Sci Rep.

[CR83] Potts SG, Vulliamy B, Dafni A, Ne'eman G, Willmer P (2003) Linking bees and flowers: How do floral rcommunities structure pollinator communities?. Ecol 84(10):2628–2642

[CR61] QGIS Development Team (2019) QGIS Geographic Information System. Version 3.6. Open Source Geospatial Foundation Project. https://www.qgis.org

[CR62] R Core Team (2020). R. A language and environment for statistical computing. Version 4.0.

[CR63] Rader R, Bartomeus I, Garibaldi LA, Garratt MPD, Howlett BG (2016). Non-bee insects are important contributors to global crop pollination. Proc Natl Acad Sci USA.

[CR64] Ricketts TH, Regetz J, Steffan-Dewenter I (2008). Landscape effects on crop pollination services: are there general patterns?. Ecol Lett.

[CR65] Riedinger V, Renner M, Rundlöf M (2014). Early mass-flowering crops mitigate pollinator dilution in late-flowering crops. Landsc Ecol.

[CR66] Rollin O, Bretagnolle V, Decourtye A (2013). Differences of floral resource use between honey bees and wild bees in an intensive farming system. Agr Ecosyst Environ.

[CR67] Rollin O, Bretagnolle V, Fortel L (2015). Habitat, spatial and temporal drivers of diversity patterns in a wild bee assemblage. Biodivers Conserv.

[CR68] Rollin O, Pérez-Méndez N, Bretagnolle V, Henry M (2019). Preserving habitat quality at local and landscape scales increases wild bee diversity in intensive farming systems. Agr Ecosyst Environ.

[CR84] Roulston T'ai H., Goodell Karen (2011). The Role of Resources and Risks in Regulating Wild Bee Populations. Annu Rev Entomol.

[CR69] Rundlöf M, Persson AS, Smith HG, Bommarco R (2014). Late-season mass-flowering red clover increases bumble bee queen and male densities. Biol Conserv.

[CR88] Schirmel Jens, Albrecht Matthias, Bauer Philipp-Martin, Sutter Louis, Pfister Sonja C., Entling Martin H., Pocock Michael (2018). Landscape complexity promotes hoverflies across different types of semi-natural habitats in farmland. Journal of Applied Ecology.

[CR70] Stoddard FL, Bond DA (1987). The pollination requirements of the faba bean. Bee World.

[CR71] Suso MJ, Moreno MT, Mondragao-Rodrigues F, Cubero JI (1996). Reproductive biology of *Vicia faba*: Role of pollination conditions. Field Crop Res.

[CR72] Sutter L, Jeanneret P, Bartual AM (2017). Enhancing plant diversity in agricultural landscapes promotes both rare bees and dominant crop-pollinating bees through complementary increase in key floral resources. J Appl Ecol.

[CR73] Sutter L, Albrecht M, Jeanneret P (2018). Landscape greening and local creation of wildflower strips and hedgerows promote multiple ecosystem services. J Appl Ecol.

[CR74] Symonds MRE, Moussalli A (2011). A brief guide to model selection, multimodel inference and model averaging in behavioural ecology using Akaike’s information criterion. Behav Ecol Sociobiol.

[CR75] Venturini EM, Drummond FA, Hoshide AK (2017). Pollination reservoirs for wild bee habitat enhancement in cropping systems: a review. Agroecol Sust Food.

[CR76] Wei T, Simko V (2017) R package “corrplot”: Visualization of a Correlation Matrix. Version 0.84 https://github.com/taiyun/corrplot

[CR77] Wenzel A, Grass I, Belavadi VV, Tscharntke T (2020). How urbanization is driving pollinator diversity and pollination – A systematic review. Biol Conserv.

[CR78] Westphal C, Steffan-Dewenter I, Tscharntke T (2003). Mass flowering crops enhance pollinator densities at a landscape scale: Flowering crops enhance pollinator densities. Ecol Lett.

[CR79] Westphal C, Steffan-Dewenter I, Tscharntke T (2009). Mass flowering oilseed rape improves early colony growth but not sexual reproduction of bumblebees. J Appl Ecol.

[CR80] Wickham H (2016) ggplot2: Elegant Graphics for Data Analysis, 2nd edn. Springer International Publishing

[CR81] Winfree R, Bartomeus I, Cariveau DP (2011). Native pollinators in anthropogenic habitats. Annu Rev Ecol Evol S.

[CR86] Wolf S, Moritz RFA (2008) Foraging distance in *Bombus terrestris* L. (Hymenoptera: Apidae). Apidologie 39(4):419–427

