# Supplementary material for: Effects of temporal floral resource availability and non-crop habitats on broad bean pollination
Source: Landsc Ecol. 2022 Apr 30;37(6):1573–86. doi: 10.1007/s10980-022-01448-2 (PMC9122849; doi:10.1007/s10980-022-01448-2)
Supplement: Supplementary file 1 — Supplementary material 1 (DOCX 1583.7 kb) [file 10980_2022_1448_MOESM1_ESM.docx]

Online Resource | Landscape Ecology

**Effects of temporal floral resource availability and non-crop habitats on broad bean pollination**

Philipp W. Eckerter^a^, Matthias Albrecht^b^, Colette Bertrand^b,c^, Erika Gobet^d^, Felix Herzog^b^, Sonja C. Pfister^e^, Willy Tinner^d^, Martin H. Entling^a^

**Affiliations:**

^a^iES Landau, Institute for Environmental Sciences, University of Koblenz-Landau, Landau (Germany)

^b^Agricultural Landscapes and Biodiversity, Agroscope, Zürich (Switzerland)

^c^Université Paris-Saclay, INRAE, AgroParisTech, UMR ECOSYS, Versailles (France)

^d^Institute of Plant Sciences and Oeschger Centre for Climate Change Research, University of Bern, Bern (Switzerland)

^e^Institute for Agroecology and Biodiversity (IFAB), Mannheim (Germany)

**Corresponding author:**

Philipp W. Eckerter
philipp.eckerter@posteo.de

Online Resource containing further information, additional figures and tables

- **A.1** Mapping details
- **A.2** Calculation of floral resource availability indices
- **Fig. A.1** Location of landscapes
- **Fig. A.2** Example set up of broad bean phytometers
- **Fig. A.3** Pearson correlation matrix between seed set and explanatory variables
- **Tab. A.1** Use and availability of key pollen types included in the study and used for calculating the resource availability index for *Bombus terrestris*
- **Tab. A.2** Pollen types other than key pollen types
- **Tab. A.3** Pearson correlation matrix between seed set and explanatory variables
- **Tab. A.4** Contribution of landscape context to floral resource availability in the landscapes
- **Tab. A.5** Comparison of seed set predictability and contributions to floral resource availability using different classifications of classical habitat maps

**A.1 Mapping details on floral resource maps**

We use cover (the projection of the crown to the ground) of plants as a proxy for the pollen amount that they offer. Assuming that the height of the canopies does not systematically differ between landscapes (averaged over hundreds to thousands of tree and shrub individuals), the cover is proportional to the volume.

We scanned each woody plant in forest edges (i.e. the first 10 m into forests) and other woody landscape elements (i.e. single standing trees, hedgerows) inside the 500 m of each landscape for its cover in m^2^ from mid-July till mid-November 2017. We calculated the tree cover inside commercial orchards by multiplying the number of rows by their mean width, a mean density factor for rows, which we measured and assessed in the field, by their mean length, which was obtained in QGIS 3.6.2. In total, we mapped around 75 ha of woody plant species.

For mapping of respective herbaceous (i.e. annual) synchronous flowering plants (*Papaver rhoeas*, *Phacelia tanacetifolia* and *Trifolium* spec.), each landscape was visited once from end-Mai till mid-July 2017. During mapping, each of these species was flowering in our study region. We obtained cover of single patchy herbaceous resources flowering on wider homogeneous areas by assessing their cover within ten 1m^2^-subplots and by extrapolating the obtained cover over the whole field area, which we calculated in QGIS.

**A.2 Calculations of floral resource availability indices**

Relative pollen type cover in a landscape:

Cover *c* of pollen type *p* in landscape *l* relative to cover *C* of pollen type *p* summed up across all landscapes:

| ${cr}_{p,l}= c_{p,l}/C_{p}$ | (Eq. 1) |
| --- | --- |

Single mean pollen type grain volume:

Single mean pollen type grain volume *vp* of pollen type *p* with *ae* (length equatorial axis) und *ap* (length polar axis; formula of spheroid):

| $v_{p}=4\pi/3 \cdot{ae}^{2}/2 \cdot{ap}_{p}/2$ | (Eq. 2) |
| --- | --- |

Measurements were taken from Beug (2004). Missing values of either *ae_p_* or *ap_p_* were calculated by PFormI (relation of length of value for available axis and highest transverse dimension of a pollen type).

Single pollen type collection volume during time period:

Single mean pollen type collection volume *cv* of a single pollen type *p* with *n* pollen grains of pollen type *p* during period *t*:

| ${cv}_{p,t}= n_{p,t} \cdot v_{p}$ | (Eq. 3) |
| --- | --- |

Total pollen type collection volume during a time period:

Total collection volume *CV* of all pollen types pooled across all landscapes during period *t*:

| ${CV}_{p,t}= \sum_{p=1}^{P} {cv}_{p,t}$ | (Eq. 4) |
| --- | --- |

Relative pollen type collection volume during a time period:

Collection volume *cv* of pollen type *p* during period *t* relative to collection volume *CV* of all pollen types during period *t*:

| ${vr}_{p, t} ={cv}_{p,t}/{CV}_{t}$ | (Eq. 5) |
| --- | --- |

Floral resource availability index:

| ${fai}_{l,t} = n\cdot\sum_{p = 1}^{P} {cr}_{p,l} \cdot{vr}_{p,t}$ | (Eq. 6) |
| --- | --- |

Floral resource availability index *fai* in landscape *l* during time period *t* is calculated by the number of landscapes *n* multiplied by sum of relative pollen type cover *cr_p_* in landscape *l* multiplied by its relative collected pollen volume *vr* of respective pollen type *p* during time period *t* and *P* gives the total number of relevant pollen types in the respective time period.


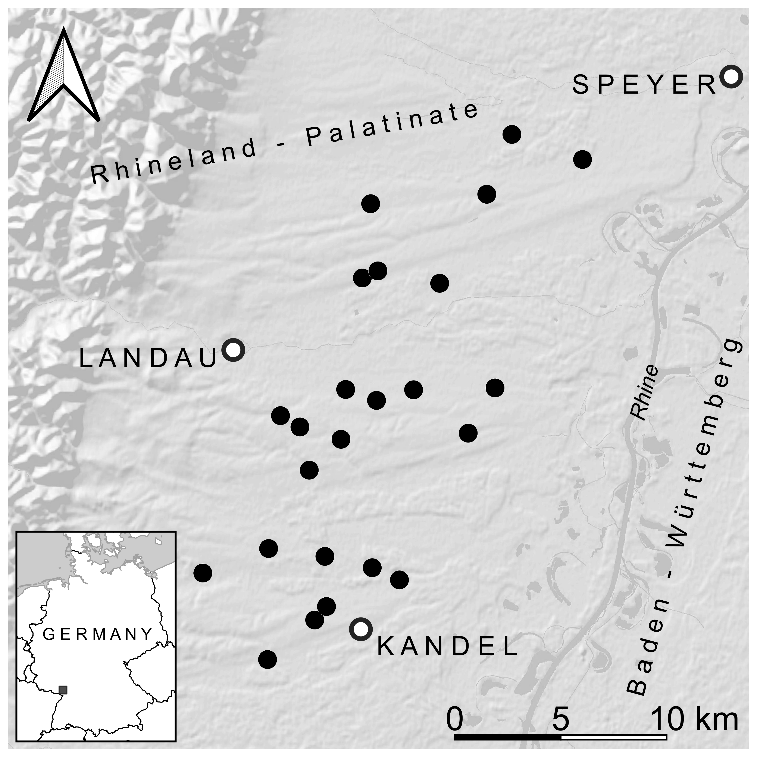


**Fig. A.1** Location of landscapes in Rhineland-Palatinate, south-west Germany


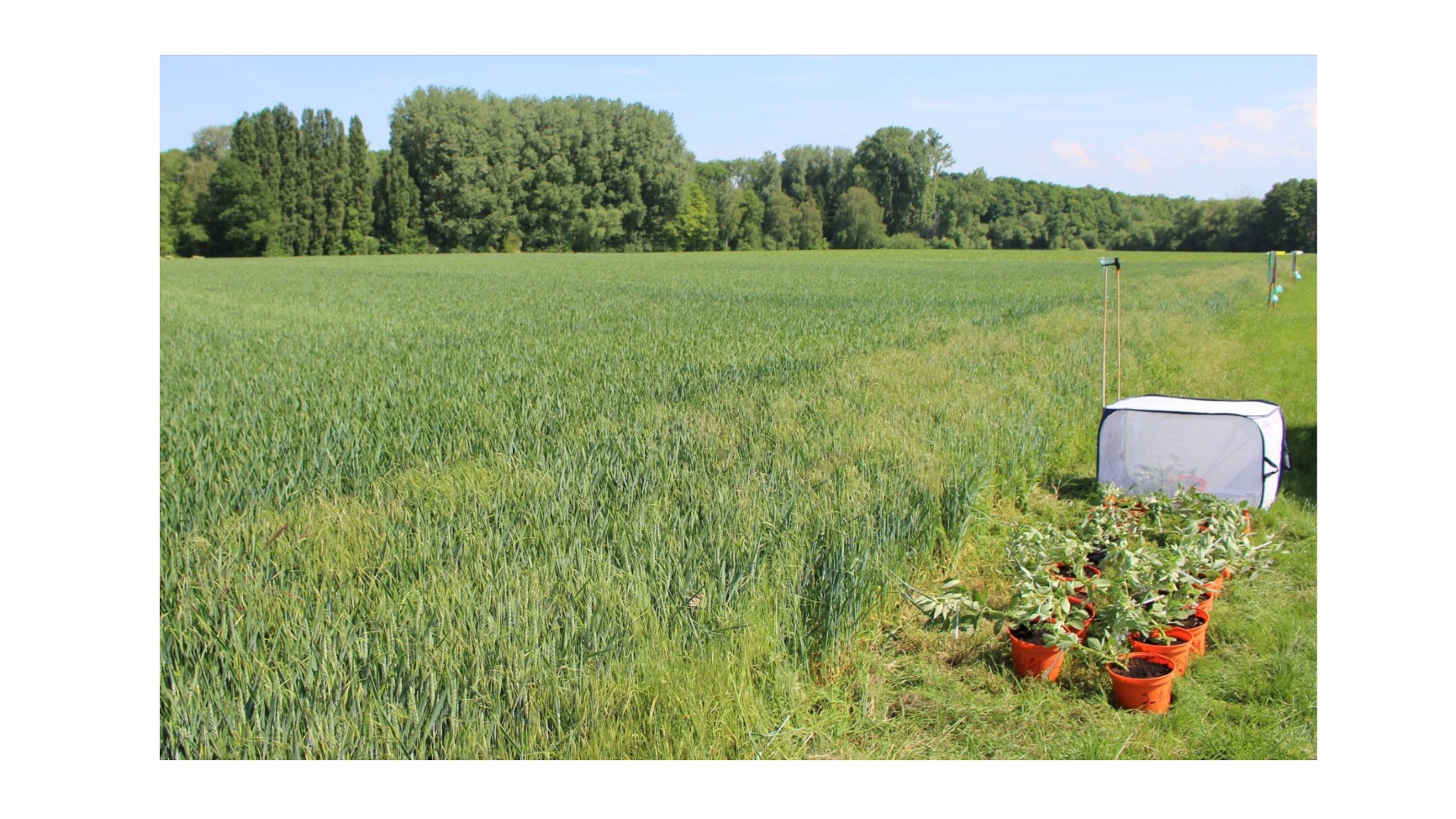


**Fig. A.2** Example set up of broad bean phytometers in a grassy field margin in the center of landscape Insheim_3.

**Table A.1** Use and availability of key pollen types included in the study and used for calculating the resource availability index for Bombus terrestris. Key pollen types are morphologically distinguishable pollen types that constitute > 5% of the collected pollen in at least one season (i.e. preceding, synchronous to broad bean flowering or across the whole season from April to July; Bertrand et al., 2019) and pollen types collected on woody plants. Plant species are species mapped in the studied landscapes that have the respective pollen morphology. Use of pollen types is given as % in the diet of Bombus terrestris during the time periods preceding and synchronous to broad bean flowering as well as pooled across the whole season. Volumes are estimated for single grains using measurements of Beug (2004) assuming spheroidal shapes of pollen grains. Index % gives the contribution of each pollen type to the resource availability index which is defined by relative cover of a pollen type proportional to the use times the relative volume (A.1, Formula 6). Vegetation type is the type of plant species associated to one pollen type (Beug 2004; w = woody, h = herbaceous), and cover is the total area of the respective plant species in the studied landscapes. Three key pollen types were excluded from the calculations: Cucurbitaceae because they were not flowering during field work of the current study, and Pterocarya and Rhamnus because they were not found in our landscapes. In total, 69 detected plant species with the respective morphology of 32 pollen types were mapped.

|  |  | ***B. terrestris* use (%)** | | |  | **Index**  **(%)** | | |  |  |
| --- | --- | --- | --- | --- | --- | --- | --- | --- | --- | --- |
| **Key pollen type** | **Plant species** | **prec.** | **sync.** | **total** | **Volume**  **(μm^3^)** | **prec.** | **sync.** | **total** | **Vegetation type** | **Cover**  **(ha)** |
| *Abies* | *A. alba, Abies* spec. | 0.08 | 0.01 | 0.08 | 803 368 | 6.1 | 0.8 | 3.0 | w | 0.382 |
| *Acer* | *A. campestre*, *A. platanoides, A. pseudoplatanus*,  *A.* spec. | 9.15 | 0.00 | 9.15 | 18 729 | 17.0 | 0.0 | 6.8 | w | 4.355 |
| *Aesculus hippocastanum* | *A. hippocastanum* | 4.11 | 0.00 | 4.11 | 6 790 | 2.8 | 0.0 | 1.1 | w | 0.107 |
| *Alnus* | *A. glutinosa, A.* spec. | 0.05 | 0.00 | 0.05 | 9 194 | 0.0 | 0.0 | 0.0 | w | 3.649 |
| *Asparagus* | *A. officinalis* | 0.00 | 5.30 | 0.00 | 5 560 | 0.0 | 3.4 | 2.0 | h | 19.539 |
| *Betula* | *B. pendula* | 1.12 | 0.00 | 1.12 | 9 795 | 1.1 | 0.0 | 0.4 | w | 0.944 |
| *Brassicaceae* | *Brassica napus* | 4.15 | 0.06 | 4.15 | 9 098 | 3.7 | 0.1 | 1.5 | h | 28.895 |
| *Carpinus betulus* | *C. betulus* | 0.25 | 0.00 | 0.25 | 32 788 | 0.8 | 0.0 | 0.3 | w | 2.290 |
| *Castanea* | *C. sativa* | 0.00 | 0.55 | 0.00 | 786 | 0.0 | 0.0 | 0.0 | w | 0.176 |
| *Cornus sanguinea* | *C. sanguinea* | 0.00 | 0.13 | 0.00 | 160 100 | 0.0 | 2.3 | 1.4 | w | 2.232 |
| *Corylus* | *C. avellana, C.* spec. | 0.02 | 0.00 | 0.02 | 12 452 | 0.0 | 0.0 | 0.0 | w | 1.545 |
| *Fagus* | *F. sylvatica* | 1.05 | 0.03 | 1.05 | 28 099 | 2.9 | 0.1 | 1.2 | w | 1.275 |
| *Ilex aquifolium* | *I. aquifolium* | 0.05 | 0.00 | 0.05 | 14 910 | 0.1 | 0.0 | 0.0 | w | 0.001 |
| *Juglans* | *J. regia* | 0.09 | 0.00 | 0.09 | 47 182 | 0.4 | 0.0 | 0.2 | w | 4.275 |
| *Ligustrum* type | *Ligustrum vulgare, Syringa vulgaris* | 0.00 | 0.07 | 0.00 | 17 726 | 0.0 | 0.1 | 0.1 | w | 0.961 |
| *Lonicera xylosteum* type | *L. xylosteum* | 0.03 | 0.00 | 0.03 | 107 493 | 0.3 | 0.0 | 0.1 | w | 0.062 |
| *Oleaceae (other)* | *Forsythia* × *intermedia* | 0.69 | 0.00 | 0.69 | 12 331 | 0.8 | 0.0 | 0.3 | w | 0.003 |
| *Papaver rhoeas* type | *P. rhoeas* | 0.00 | 13.0 | 0.00 | 9 120 | 0.0 | 13.6 | 8.1 | h | 0.012 |
| *Phacelia tanacetifolia* | *P. tanacetifolia* | 0.02 | 6.46 | 0.02 | 3 292 | 0.0 | 2.4 | 1.5 | h | 0.070 |
| *Picea* | *Picea* spec. | 0.00 | 0.02 | 0.00 | 659 710 | 0.0 | 1.4 | 0.8 | w | 0.601 |
| *Pinus* | *Pinus* spec. | 0.09 | 0.00 | 0.09 | 118 024 | 1.1 | 0.0 | 0.4 | w | 0.574 |
| *Potentilla* type | *Fragaria* spec. only | 4.54 | 0.00 | 4.54 | 4 374 | 2.0 | 0.0 | 0.8 | h | 2.082 |
| *Prunus* type | *Amelanchier ovalis, Crataegus* spec.*, Cydonia oblonga*, *Malus domestica, Malus sylvestris, Malus* spec., *Mespilus germanica, Prunus armeniaca, P. avium,  P. cerasus, P. domestica, P. domestica* subsp. *Syriaca, P. dulcis, P. laurocerasus, P. padus, P. persica,  P. serotina, P. spinosa, P.* spec.*, Pyrus communis, Pyrus domestica, Pyrus pyraster, Pyrus* spec. | 21.2 | 0.00 | 21.2 | 23 810 | 50.1 | 0.0 | 20.2 | w | 21.565 |
| *Quercus* | *Q. alba, Q. petraea, Q. robur, Q. rubra, Q. spec.* | 0.69 | 0.00 | 0.69 | 14 796 | 1.0 | 0.0 | 0.4 | w | 3.727 |
| *Robinia pseudoacacia* | *R. pseudoacacia* | 0.00 | 1.10 | 0.00 | 15 862 | 0.0 | 2.0 | 1.2 | w | 1.634 |
| *Rosaceae* | *R. agrestis, R. canina, R.* spec., *Sorbus aucuparia, Sorbus domestica, Sorbus* spec. | 1.66 | 5.14 | 1.66 | 10 114 | 1.7 | 6.0 | 4.2 | w | 0.967 |
| *Rubus* type | *R. fruticosus, R. idaeus* | 0.00 | 23.8 | 0.00 | 11 599 | 0.0 | 31.7 | 19.0 | w | 4.262 |
| *Salix* | *S. alba, S. babylonica, S. caprea, S. fragilis,  S. matsudana ‘Tortuosa’, S.* spec. | 37.1 | 0.35 | 37.1 | 2 157 | 7.9 | 0.1 | 3.2 | w | 6.870 |
| *Sambucus nigra* type | *S. nigra, S. racemosa, S.* spec. | 0.11 | 0.13 | 0.11 | 2 405 | 0.0 | 0.0 | 0.0 | w | 1.808 |
| *Tilia* | *T. chordata, T. platyphyllos* | 0.02 | 20.5 | 0.02 | 13 196 | 0.0 | 4.9 | 2.9 | w | 0.146 |
| *Trifolium repens* type | *T. repens* | 0.03 | 5.04 | 0.03 | 8 476 | 0.0 | 31.0 | 18.5 | h | 0.554 |
| *Viburnum* | *V. lantana, V. opulus* | 0.00 | 0.04 | 0.00 | 7 645 | 0.0 | 0.0 | 0.0 | w | 0.220 |
| **32 pollen types** | **69 plant species** | **86.3** | **81.7** | **83.7** |  | **100** | **100** | **100** |  | **115.8** |

**Tab. A.2** Pollen types other than key pollen types are morphologically distinguishable pollen types that constitute < 5% of the collected pollen in at least one season (i.e. preceding, synchronous to broad bean flowering or across the whole season from April to July; Bertrand et al., 2019). Use of pollen types is given as % in the diet of Bombus terrestris during the time periods preceding and synchronous to broad bean flowering as well as pooled across the whole season. Vegetation type is the type of plant species associated to one pollen type (Beug, 2004; w = woody, h = herbaceous).

|  | ***B. terrestris* use (%)** | | |  |
| --- | --- | --- | --- | --- |
| **Pollen type** | **prec.** | **sync.** | **total** | **Vegetation type** |
| *Achillea* type | 0.00 | 0.07 | 0.0 | h |
| *Allium* type | 0.00 | 0.01 | 0.0 | h |
| *Apiaceae* | 0.00 | 0.79 | 0.5 | h |
| *Campanula* type | 0.78 | 0.00 | 0.3 | h |
| *Campanulaceae* | 0.00 | 0.01 | 0.0 | h |
| *Caryophyllaceae* | 0.09 | 0.00 | 0.0 | h |
| *Centaurea cyanus* type | 0.00 | 0.04 | 0.0 | h |
| *Centaurea jacea* type | 0.00 | 0.10 | 0.1 | h |
| *Cerealia* type | 0.38 | 0.00 | 0.1 | h |
| *Chelidonium majus* | 0.26 | 0.00 | 0.1 | h |
| *Chenopodiaceae* | 1.17 | 0.00 | 0.4 | h |
| *Cichorioideae* | 1.74 | 0.04 | 0.7 | h |
| *Corydalis* | 0.02 | 0.00 | 0.0 | h |
| *Cucurbita* | 0.00 | 0.74 | 0.5 | h |
| *Echium* | 0.26 | 0.00 | 0.1 | h |
| *Galium* type | 0.00 | 0.02 | 0.0 | h |
| *Genista* type | 1.11 | 0.04 | 0.4 | h |
| *Helianthemum* | 0.00 | 0.74 | 0.5 | h |
| *Hypericum perforatum* type | 0.00 | 3.51 | 2.2 | h |
| *Lamium* type | 4.65 | 0.00 | 1.7 | h |
| *Malvaceae* | 0.00 | 0.01 | 0.0 | h |
| *Mentha* type | 0.22 | 1.00 | 0.7 | h |
| *ND (pollen grains could not be identified)* | 0.00 | 3.08 | 1.9 | NA |
| *Plantago lanceolata* type | 0.00 | 0.01 | 0.0 | h |
| *Poaceae* | 0.34 | 0.15 | 0.2 | h |
| *Potentilla* type (other than *Fragaria* spec.) | 0.00 | 2.23 | 1.1 | h |
| *Pterocarya* | 0.02 | 0.00 | 0.0 | w |
| *Ranunculus acris* type | 0.12 | 0.15 | 0.1 | h |
| *Reseda* | 0.00 | 0.84 | 0.5 | h |
| *Rhamnus* type | 0.00 | 1.04 | 0.7 | w |
| *Ribes* | 0.98 | 0.05 | 0.4 | h |
| *Rinanthus* type | 1.26 | 0.04 | 0.5 | h |
| *Sedum* type | 0.00 | 1.61 | 1.0 | h |
| *Silene* type | 0.23 | 0.00 | 0.1 | h |
| *Solanum dulcamara* | 0.00 | 0.04 | 0.0 | h |
| *Stachys sylvatica* type | 0.02 | 0.00 | 0.0 | h |
| *Thalictrum* | 0.00 | 0.04 | 0.0 | h |
| *Trifolium pratense* type | 0.02 | 0.89 | 0.6 | h |
| *Urtica* | 0.00 | 0.09 | 0.1 | h |
| *Vicia* type | 0.00 | 0.90 | 0.6 | h |
| **39 pollen types + ND** | **13.7** | **18.3** | **16.3** |  |

**Tab. A.3** Pearson correlation matrix between seed set (seeds per pod) and predictors using different mapping approaches. Floral resource maps are represented by floral resource availability preceding and synchronous to broad bean flowering as well as pooled across the whole season from April to July (total), classical habitat maps by landscape variables (proportions of arable land, permanent crops, forest, other woody and herbaceous semi-natural habitat and urban, as well as its Euclidean distances). For visualisation of the correlation matrix see Fig. A.4

|  | **seed set** |  | **floral resource maps** | | |  | **classical habitat maps** | | | | | | | | |
| --- | --- | --- | --- | --- | --- | --- | --- | --- | --- | --- | --- | --- | --- | --- | --- |
|  | **sp** |  | **prec.** | **sync.** | **total** |  | **arable** | **crop permanent** | **forest** | **snh herb** | **snh woody** | **urban** | **dist forest** | **dist snh herb** | **dist snh woody** |
| **prec.** | 0.42^c^ |  |  |  |  |  |  |  |  |  |  |  |  |  |  |
| **sync.** | -0.06 |  | 0.41^c^ |  |  |  |  |  |  |  |  |  |  |  |  |
| **total** | 0.16 |  | 0.76^a^ | 0.90^a^ |  |  |  |  |  |  |  |  |  |  |  |
| **arable** | -0.42^c^ |  | -0.60^b^ | -0.52^b^ | -0.65^a^ |  |  |  |  |  |  |  |  |  |  |
| **crop permanent** | 0.24 |  | 0.43^c^ | 0.11 | 0.27 |  | -0.26 |  |  |  |  |  |  |  |  |
| **forest** | 0.26 |  | 0.40^d^ | 0.55^b^ | 0.59^b^ |  | -0.70^a^ | -0.02 |  |  |  |  |  |  |  |
| **snh herb** | 0.14 |  | 0.32 | 0.23 | 0.45^c^ |  | -0.74^a^ | 0.01 | 0.20 |  |  |  |  |  |  |
| **snh woody** | 0.34 |  | 0.41 | 0.35^d^ | 0.42^c^ |  | -0.78^a^ | 0.12 | 0.37 | 0.65^a^ |  |  |  |  |  |
| **urban** | 0.46^c^ |  | 0.13 | 0.08 | 0.11 |  | -0.41^c^ | -0.23 | 0.01 | 0.40^d^ | 0.51^c^ |  |  |  |  |
| **dist forest** | -0.19 |  | -0.23 | -0.55^b^ | -0.50^c^ |  | 0.49^c^ | 0.11 | -0.45^c^ | -0.28 | -0.56^b^ | -0.29 |  |  |  |
| **dist snh herb** | -0.03 |  | -0.15 | -0.26 | -0.24 |  | 0.31 | -0.17 | -0.20 | -0.19 | -0.29 | -0.09 | 0.10 |  |  |
| **dist snh woody** | 0.05 |  | -0.03 | -0.29 | -0.18 |  | 0.29 | 0.06 | 0.04 | -0.33 | -0.58^b^ | -0.27 | 0.41^c^ | -0.08 |  |
| **dist urban** | -0.20 |  | -0.17 | 0.12 | 0.01 |  | 0.38^d^ | -0.01 | -0.07 | -0.37^d^ | -0.35^d^ | -0.54^b^ | 0.15 | 0.16 | 0.26 |

Notes: correlation is significant at the ^a^ 0.001-level; ^b^ 0.01-level; ^c^ 0.05-level; ^d^ 0.10-level.

**
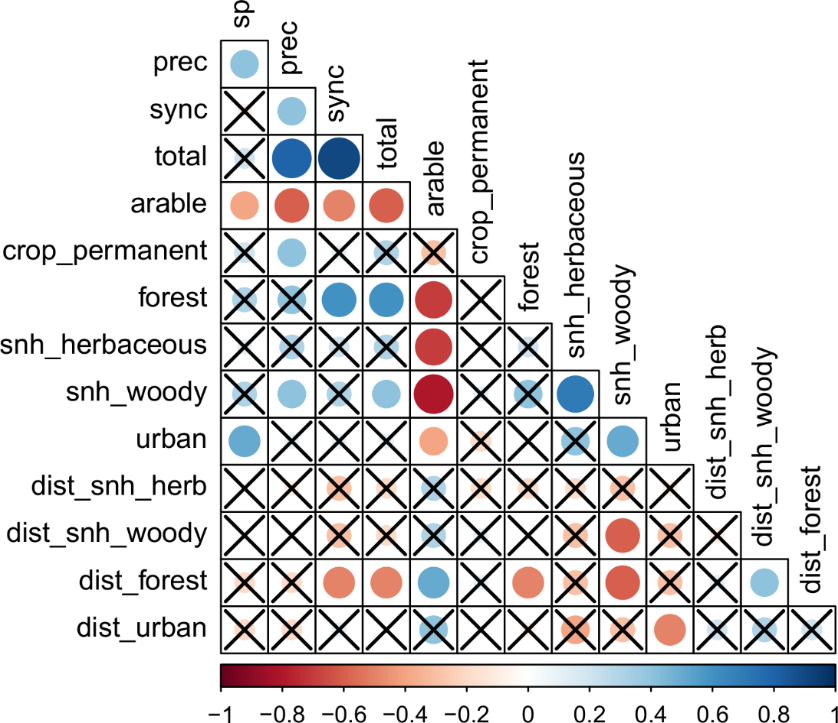
**

**Fig. A.3** Pearson correlation matrix between seed set (seeds per pod) and predictors using different mapping approaches. Floral resource maps are represented by floral resource availability preceding and synchronous to broad bean flowering as well as pooled across the whole season from April to July (total), classical habitat maps by landscape variables (proportions of arable land, permanent crops, forest, other woody and herbaceous semi-natural habitat and urban, as well as its Euclidean distances). An “X” was plotted over circles representing non-significant correlations (i.e. p ≥ 0.05). For relating r values see Tab. A.4. The matrix was drawn using the corrplot package in R (Wei and Simko 2017).

**Tab. A.4** Contribution of landscape context to floral resource availability in the landscapes during time periods preceding and synchronous to broad bean flowering and across the whole season from April to July using linear regression.

| **Floral resources** | **Predictor** | **df** | **t** | ***R^2^_mult_*** | **p value** |
| --- | --- | --- | --- | --- | --- |
| Preceding | **Arable** | **22** | **-2.05** | **0.364** | **0.002** |
|  | **Permanent crop** | **22** | **2.20** | **0.181** | **0.038** |
|  | Forest | 22 | 2.04 | 0.159 | 0.054 |
|  | Herbaceous SNH | 22 | 1.57 | 0.101 | 0.130 |
|  | **Woody SNH** | **22** | **2.11** | **0.168** | **0.047** |
|  | Urban | 22 | 0.62 | 0.017 | 0.540 |
|  | Distance forest | 22 | -1.11 | 0.053 | 0.281 |
|  | Distance herbaceous SNH | 22 | -0.69 | 0.021 | 0.495 |
|  | Distance woody SNH | 22 | 0.13 | 0.001 | 0.900 |
|  | Distance urban | 22 | -0.80 | 0.028 | 0.433 |
| Synchronous | **Arable** | **22** | **-2.88** | **0.274** | **0.009** |
|  | Permanent crop | 22 | 0.52 | 0.012 | 0.605 |
|  | **Forest** | **22** | **3.12** | **0.307** | **0.005** |
|  | Herbaceous SNH | 22 | 1.10 | 0.052 | 0.285 |
|  | Woody SNH | 22 | 1.73 | 0.120 | 0.097 |
|  | Urban | 22 | 0.39 | 0.007 | 0.698 |
|  | **Distance forest** | **22** | **-3.08** | **0.301** | **0.006** |
|  | Distance herbaceous SNH | 22 | -1.26 | 0.067 | 0.222 |
|  | Distance woody SNH | 22 | -1.43 | 0.085 | 0.168 |
|  | Distance urban | 22 | 0.58 | 0.015 | 0.571 |
| Total | **Arable** | **22** | **-4.01** | **0.422** | **0.001** |
|  | Permanent crop | 22 | 1.33 | 0.074 | 0.198 |
|  | **Forest** | **22** | **3.43** | **0.349** | **0.002** |
|  | Herbaceous SNH | 22 | 1.48 | 0.090 | 0.154 |
|  | **Woody SNH** | **22** | **2.19** | **0.180** | **0.039** |
|  | Urban | 22 | 0.52 | 0.012 | 0.606 |
|  | **Distance forest** | **22** | **-2.69** | **0.248** | **0.013** |
|  | Distance herbaceous SNH | 22 | -1.19 | 0.060 | 0.247 |
|  | Distance woody SNH | 22 | -0.84 | 0.031 | 0.413 |
|  | Distance urban | 22 | 0.07 | 0.000 | 0.947 |

**Tab. A.5** Comparison of seed set predictability and contributions of landscape context to floral resource availability preceding, synchronous to broad bean flowering and across the whole season from April to July using broader and finer classifications of classical habitat maps and linear regression. Significant relations (i.e. p < 0.05) are printed in bold.

| **Response** | **Classification type** | **Predictor** | **df** | **t** | ***R^2^_mult_*** | **p value** |
| --- | --- | --- | --- | --- | --- | --- |
| Seed set | Broad | Crop | 22 | -1.89 | 0.140 | 0.072 |
|  |  | SNH | 22 | 1.34 | 0.076 | 0.193 |
|  | Fine | **Arable** | **22** | **-2.19** | **0.179** | **0.039** |
|  |  | Permanent crops | 22 | 1.14 | 0.055 | 0.269 |
|  |  | Forest | 22 | 1.25 | 0.066 | 0.225 |
|  |  | Herbaceous SNH | 22 | 0.67 | 0.020 | 0.509 |
|  |  | Woody SNH | 22 | 1.71 | 0.117 | 0.102 |
| Preceding | Broad | Crop | 22 | -2.77 | 0.259 | 0.011 |
| floral |  | SNH | 22 | 2.52 | 0.224 | 0.020 |
| resources | Fine | **Arable** | **22** | **-3.55** | **0.364** | **0.002** |
|  |  | **Permanent crops** | **22** | **2.20** | **0.181** | **0.038** |
|  |  | Forest | 22 | 2.04 | 0.159 | 0.054 |
|  |  | Herbaceous SNH | 22 | 1.57 | 0.101 | 0.130 |
|  |  | **Woody SNH** | **22** | **2.11** | **0.168** | **0.047** |
| Synchronous | Broad | **Crop** | **22** | **-2.79** | **0.261** | **0.011** |
| floral |  | **SNH** | **22** | **2.82** | **0.266** | **0.010** |
| resources | Fine | **Arable** | **22** | **-2.88** | **0.274** | **0.009** |
|  |  | Permanent crops | 22 | 0.52 | 0.012 | 0.605 |
|  |  | **Forest** | **22** | **3.12** | **0.307** | **0.005** |
|  |  | Herbaceous SNH | 22 | 1.10 | 0.052 | 0.285 |
|  |  | Woody SNH | 22 | 1.73 | 0.120 | 0.097 |
| Total | Broad | **Crop** | **22** | **-3.50** | **0.357** | **0.002** |
| floral |  | **SNH** | **22** | **3.40** | **0.345** | **0.003** |
| resources | Fine | **Arable** | **22** | **-4.01** | **0.422** | **0.001** |
|  |  | Permanent crops | 22 | 1.33 | 0.074 | 0.198 |
|  |  | **Forest** | **22** | **3.43** | **0.349** | **0.002** |
|  |  | Herbaceous SNH | 22 | 1.48 | 0.090 | 0.154 |
|  |  | **Woody SNH** | **22** | **2.19** | **0.180** | **0.039** |

**References**

Bertrand C, Eckerter PW, Ammann L, et al (2019) Seasonal shifts and complementary use of pollen sources by two bees, a lacewing and a ladybeetle species in European agricultural landscapes. J Appl Ecol 56:2431–2442. https://doi.org/10.1111/1365-2664.13483

Beug H-J (2004) Leitfaden der Pollenbestimmung für Mitteleuropa und angrenzende Gebiete. Verlag Dr. Friedrich Pfeil, München

Wei T, Simko V (2017) R package “corrplot”: Visualization of a Correlation Matrix. Version 0.84URL https://github.com/taiyun/corrplot
